# Announcement

**DOI:** 10.1155/S1463924699000188

**Published:** 1999


PS Analytical has also developed the Sir Galahad II instrument which is a market leader for the analysis of mercury in the National Gas Industry. It can be used in both on-line and off-line modes.

### References

*Methods for the Examination of Waters and Associated Materials*

*Mercury in Waters, Effluents, Soils and Sediments,* etc., *Additional Methods* (1985). Published by Her Majesty's Stationery Office, London, UK.

*For further information on these and other PS Analytical products, either visit our Website at http://www.psanalytical.demon.co.uk or contact us at PS Analytical Ltd, Arthur House, Crayfields Industrial Estate, Main Road, Orpington, Kent, BR5 3HP, UK. Tel (Int): +44 (0)1689 891211; Fax (Int): +44 (0)1689 896009; e-mail: psa@psanalytical.demon.co.uk*

### Shimadzu technique for food safety testing by analysis of plastic packaging

Printed plastic film—a common packaging material for many food products—contains solvent residues, e.g. alcohols, hydrocarbons and ketones which, because of their high volatility, can be easily absorbed by food and then by the people who eat it.

The main problem for food and packaging materials manufacturers has been the accurate quantification of components in packaging material so that leaching of solvent to foodstuffs can be minimized, and optimum hygeine and health and safety standards attained.

The headspace analysis method using a Shimadzu gas chromatograph CG-14A and HSS-2B headspace system is a particularly useful technique for this; its easy, user-friendly handling system and high level of automation make it a fast, accurate and efficient approach to the routine determination of the solvent content of packaging materials.

Quantification is possible via conventional external calibration, or by standard addition techniques where solutions of different concentrations are added to the sample for analysis.

In all tests using the Shimadzu headspace methodology and instruments, the approach has been found to yield consistently accurate and sensitive results in the qualitative and quantitative analysis of volatile solvents in printed plastic film, giving analysts a fast and reliable technique for greater confidence in food safety and hygiene.

*For more details on Shimadzu headspace technology—including free copies of scientific application notes describing the technique in use—and information on other Shimadzu products for foodstuff and drinks manufacturing and processing, contact Shimadzu on +44 (0)1908 552200 or e-mail Sales@Shimadzu.co.uk*

*For more information, please contact: Shimadzu UK, Mill court, Featherstone Road, Wolverton Mill South, Milton Keynes MK12 5RE UK; Tel.: +44 (0)1908 552200; Fax.: +44 (0)1908 552211; e-mail: Sales@Shimadzu.co.uk or Ross Heaven, Strong Words, 32 Cranstoun Street, Northampton NN1 3BH, UK; Tel/Fax: +44 (0)1604 250221; e-mail: rossheaven @aol.com*

### Announcement

### ISLAR '99—Keynote Talks by Novartis and Roche Biosciences Highlight the 17th International Symposium on Laboratory Automation and Robotics

ISLAR '99, the seventeenth in a continuing series of symposia dedicated to laboratory automation and robotics, will be held at the Boston Park Plaza Hotel during 17–20 October 1999. A strong emphasis will be placed on presentations by senior laboratory managers and on applications in drug discovery research. The preliminary programme details many of the presentations and session topics as well as short courses and interactive discussion groups. Visit the ISLAR Web pages for more information, including abstracts of the presentations (http://www.islar.com).

The opening plenary session will feature two keynote speakers. Stefanie G. Nair PhD, Head of Quality Assurance at Novartis Pharmaceuticals Corporation, will speak on 'Working smarter: automation as a strategy for laboratory efficiency objectives'. Roger L. Whiting PhD, Senior Vice President of Roche Bioscience, will discuss 'Frontiers in drug discovery: use of new technologies'.

Parallel podium presentations and afternoon poster sessions will cover a variety of automation topics in the following areas:

- drug discovery
- pharmaceutical development and quality control
- bioanalytical research
- chemical and related industries
- managing laboratory automation
- emerging technologies for combinatorial chemistry
- compound preparation and distribution
- high throughput structural characterization
- high throughput screening

- ultrahigh throughput screening and assay miniaturization
- automation technologies for genomics
- secondary screening
- assay optimization and methods development
- data management/data handling and bioinformatics
- introduction to laboratory robotics
- automation strategies for assay development and primary and secondary screening
- dissolution testing: theory, practice and automation techniques
- bioanalytical automation: trends, technology and new developments
- EasyLab® programming: tips and tricks
- PCS™: tips and tricks

The three-day programme consists of over 120 podium and poster presentations describing laboratory robotics and laboratory workstation procedures from pharmaceutical, biotechnology and chemical laboratories. The symposium's technical presentations and informal discussions provide an excellent opportunity for laboratory managers, automation users and novices to exchange ideas and applications experience.

*For more information write or call: Christine O'Neil, ISLAR, 68 Elm Street, Hopkinton, MA 01748, USA. Tel: +1 508 497 2224, Fax: +1 508 435 3439, e-mail: islar@islar.com, URL—http://www.islar.com*

### Meeting report

### ISLAR '98 Recognizes Pioneers in Laboratory Automation and Robotics

The 16th International Symposium on Laboratory Automation and Robotics, held at the Boston Park Plaza Hotel during 18–21 October, recognized six scientists for their roles in developing new applications and techniques, designing unique solutions to technical problems, and implementing laboratory automation in their organizations. Nominated by colleagues in their companies or industry, these scientists were selected for this recognition by a committee of previous award winners.

The six scientists included:

Muhammed Alburakeh, Barr Laboratories, Pomona, NY, USA;
Siegfried Brandtner, Grunenthal GmbH, Germany;
Harnath Doddapaneni, SmithKline Beecham Pharmaceuticals, King of Prussia, PA;
Jim Perry, Procter & Gamble, UK;
Richard Wildonger, Zeneca Pharmaceuticals, Wilmington, DE, USA;
Rudy Willebrords, Janssen Research Foundation, Belgium.

ISLAR '98 attracted about 700 scientists from 33 states and 19 countries for three days of presentations on laboratory automation and robotics. The premier technical symposium in this field featured a program with nearly 150 papers and posters, seven discussion groups and several workshops, and six hands-on short courses, including the following areas of interest.

- High throughput screening.
- Data management/data handling.
- Ultra high-throughput screening.
- Managing laboratory automation.
- Methods transfer/validation.
- Pharmaceutical analysis.
- Bioanalytical applications.
- Advanced topics.
- Automated synthesis.
- Compound purification, distribution and analysis in drug discovery.
- Managing pharmaceutical development and QC laboratories.
- Automated physical testing & chemical analysis.
- Consumer products/food applications.
- Predictive design/structural characterization.

*For more information contact: Christine O'Neil, ISLAR, 68 Elm Street, Hopkinton, MA 01748, USA; Tel.: +1 508/435-9500; Fax.: +1 508/435-3439; e-mail: islar@islar.com*

